# ATG101-related signature predicts prognosis and therapeutic option in hepatocellular carcinoma

**DOI:** 10.1038/s41598-022-22505-5

**Published:** 2022-10-27

**Authors:** Bin Wang, Jiantao Fu, Yuanji Lin, Yi Lou, Anqian Lu, Jin Yang

**Affiliations:** 1 Department of Hepatobiliary Surgery, Affiliated Dongyang Hospital of Wenzhou Medical University, Dongyang, 322100 Zhejiang People’s Republic of China; 2 Department of Translational Medicine Center, Affiliated Hospital of Hangzhou Normal University, Hangzhou, 310015 Zhejiang People’s Republic of China; 3 Institute of Hepatology and Metabolic Diseases, Hangzhou Normal University, Hangzhou, 310015 Zhejiang People’s Republic of China; 4 Department of Research, Hangzhou MC Life Sciences Co., Ltd, Hangzhou, 311500 Zhejiang People’s Republic of China; 5 Department of Occupational Medicine, Zhejiang Provincial Integrated Chinese and Western Medicine Hospital, Hangzhou, 310003 People’s Republic of China

**Keywords:** Biomarkers, Oncology, Risk factors

## Abstract

Autophagy plays a critical role in tumor pathogenesis. However, autophagy-related signature in Hepatocellular carcinoma (HCC) has not been revealed yet. We quantified the levels of various cancer hallmarks and identified ATG101 as the major risk factor for overall survival in HCC. A robust ATG101-related gene signature (ATS) for prognosis was constructed using a combination of bioinformatic and statistical approaches. Additionally, genetic and immunological properties were measured between ATS-high and ATS-low groups. The ATS signature was associated with shortened overall survival in HCC patients independently of clinicopathological characteristics. ATS status defines an inflamed yet exhausted tumor microenvironment, in which the activities of the exhausted CD8+ or CD4+ T cells were strongly associated with ATS. The ATS signature predicts the drug resistance to the immunotherapy, thus a combination of targeted therapy and immunotherapy might be suitable for ATS-high patients. This work shed light on the function of ATG101-related genes in HCC and revealed that the ATS signature may be a useful prognostic biomarker for differentiating molecular and immunological features and predicting probable response to the therapy.

## Introduction

Hepatocellular carcinoma (HCC) is one of the most prevalent malignant solid tumors, accounting for the fourth highest number of cancer-related fatalities worldwide^1^. Despite the advancement of chemotherapy, radiation, immunotherapy, and liver transplantation, the prognosis of HCC remains poor owing to the high risk of recurrence and metastasis^2^. Thus, better prediction indicators are urgently needed to reliably estimate the prognosis of HCC patients.

Several prior research have developed prognostic models for HCC based on criteria such as clinical baseline features and molecular biomarkers^3^. Single gene aberrant expression levels, such as SEC14L2^4^, FMO4^5^, PRPF3^6^, AGBL2^7^, have been described as predictive markers for HCC patients. Furthermore, a risk coefficient model based on a multigene mRNA expression signature has been found as an independent predictive factor for overall survival (OS), with the ability to stratify patients into high- and low-risk groups with substantially different overall survival (OS)^8–10^. Due to the complexity and heterogeneity of the disease, additional gene signatures are required for accurate prediction of HCC.

Autophagy is critical for the breakdown of damaged organelles and old proteins, as well as maintaining cellular homeostasis^11^. In cancer biology, autophagy has a dual function either promoting or suppressing tumor growth^12^. Autophagy-related protein 101 (ATG101) is a new autophagy factor that is essential for autophagosome formation^13^. In cancer cells, knocking down ATG101 caused significant growth retardation and reduced survival under nutritional deprivation^14^. However, the function of ATG101 in HCC has yet to be determined.

In this work, we combined multiple computational methods to create an ATG101-related gene signature to predict prognosis and immune checkpoint blockade (ICB) therapeutic responsiveness in HCC patients, and also discussed the biological implication of this signature.

## Methods

### Data preparation and processing

The mRNA expression data [level 3; fragment per kilobase million (FPKM) normalized] from 374 tumor samples and 50 adjacent normal samples with corresponding clinicopathological information was downloaded from The Cancer Genome Atlas (TCGA) database (https://portal.gdc.cancer.gov/). RNA-seq data and clinical information of another HCC cohort were obtained from the International Cancer Genome Consortium (ICGC) (https://dcc.icgc.org/projects/LIRI-JP). After cleaning data, a total of 371 HCC patients in the TCGA database were included in the training cohort, and 212 patients in the ICGC database were included in the validation cohort. FPKM values were converted into TPM (Transcripts per million) format and log2 transformed.

### Collection of Somatic Alteration Data

Mutation data, which are sorted in the form of Mutation Annotation Format (MAF), were obtained from the cBioPortal for Cancer Genomics^15^, and analysed using R package ‘maftools’^16^. Mutually exclusive or co-occurring set of genes were examined using somaticInteractions function, which performs pair-wise Fisher’s Exact test to detect such significant pair of genes^16^.

### Candidate selection and signature establishment

The gene sets of the cancer hallmark came from the CancerSEA database (http://biocc.hrbmu.edu.cn/CancerSEA/), which aims to decipher unique functional states of cancer cells at single-cell resolution^17^. The pathway gene sets of "c2.cp.v7.0.symbols.gmt" were retrieved from the Molecular Signature Database (MSigDB). Signature gene sets for each subtype of infiltrating T cells in liver cancer were derived from single-cell sequencing, as described by Zheng et al.^18^. The score of gene set was assessed using single-sample gene set enrichment analysis (ssGSEA)^19^.

The weighted gene co-expression network analysis (wgcna) was adopted to build a scale-free co-expression network^20^. A pairwise Pearson correlation coefficient matrix was first computed, followed by an adjacency matrix and topological overlap matrix (TOM) constructed. Modules were identified on the dendrogram using the dynamic tree cut algorithm. The module with the highest correlation with ATG101 scores was calculated based on the pearson correlation. A gene with high intramodular connectivity (K.in) in a module was considered to be a hub gene^21^.

To construct the gene signature based on the above 51 hub genes (K.in > 50), a least absolute shrinkage and selection operator (LASSO)-Cox regression model was used for variable screening and dimensionality reduction. The penalty value parameter was computed using the ‘glmnet’ package after a 100-fold cross-validation. An ATG101-related risk score (ATS) was established by including normalized gene expression values weighted by their LASSO-Cox coefficients as follows: ATS = ∑_i_ Coefficient (mRNA_i_) × Expression (mRNA_i_).

### Comprehensive analysis of molecular characteristics of ATS

The differential expression genes (DEGs) between the high- and low-ATS groups were determined using the ‘Limma’ package^22^. Functional annotations were performed by using Kyoto Encyclopedia of Genes and Genomes (KEGG) pathways^23^. Tumor immune microenvironment (TIME) was estimated by CIBERSORT (http://cibersort.stanford.edu/)^24^ or the xCell algorithm (https://xcell.ucsf.edu/)^25^. The Tumor Immune Single-cell Hub (TISCH, http://tisch.comp-genomics.org/)^26^, which offers single-cell level cell-type annotation, was used to assess the ATS signature in each subgroup of immune cells. The T cell inflamed score (TIS) was calculated as a weighted linear combination of the 18 genes to define pre-existing cancer immunity^27^. A list of immunomodulatory genes including chemokines, cytokines, receptors, MHC and immune stimulators, inhibitory immune checkpoints was curated from the previous study^28^.

### Chemotherapeutic and immunotherapeutic response prediction

The Computational Analysis of Resistance (CARE, http://care.dfci.harvard.edu/)^29^ was used to identify genes related with treatment effectiveness using the Cancer Therapeutics Response Portal (CTRP), Cancer Cell Line Encyclopedia (CCLE), and the Genomics of Drug Sensitivity in Cancer databases (GDSC)^30^. A positive CARE score implies that a greater expression value is related with improved therapeutic effect, while a negative score suggests increased drug resistance.

The TIDE (Tumor Immune Dysfunction and Exclusion) algorithm (http://tide.dfci.harvard.edu/) was used to predict immunotherapy response^31^. The IC50 (half-maximal inhibitory concentration) values of HCC patients were determined by the pRRophetic package to estimate drug sensitivity^32^.

### Statistical analysis

All statistical analyses were conducted using the R version 3.6.1 software package (http://www.r-project.org). Continuous variables were compared using Wilcoxon tests. OS, Progression Free Survival (PFI), Disease Specific Survival (DSS), and Disease Free Survival (DFS) were defined according to the criteria as previously described^33^. The log-rank test was used to determine if there were significant differences in Kaplan–Meier (KM) curves. The survivalROC package was employed to plot the time-dependent ROC curve to determine the prognostic value of the signature. The nomogram, calibration curve (rms package), and decision curve analysis (DCA) were used to assess the accuracy of the prognostic model. All statistical tests were two-sided, and a significance level of p < 0.05 was used.

## Results

### Schematic diagram of the study design

**Figure 1:**
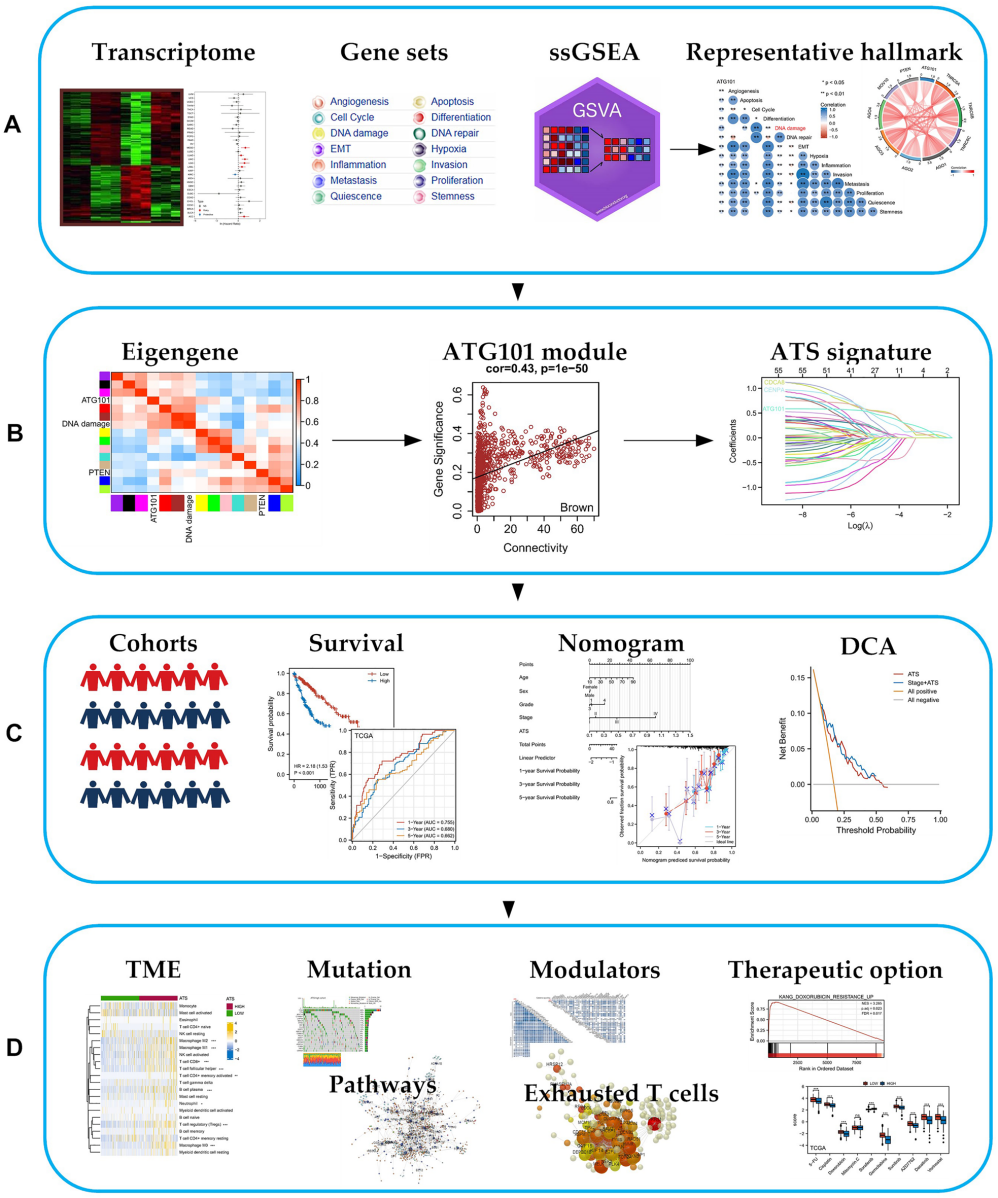
Schematic diagram of the study design. (**A**) A summary of the data sources used in the study to generate the gene signatures. ATG101 was identified as an oncogene in HCC after pan-cancer screening. (**B**) ATG101-related gene signature (ATS) was established using a combination of approaches, such as WGCNA and LASSO-Cox regression. (**C**) The performance of ATS was validated in external cohorts, using time-dependent receiver operating characteristic, nomogram and DCA. (**D**) Characterization of the feature of ATS, including its effect on TME, mutation profile, immunological properties and therapeutic implications.

After pan-cancer analysis, ATG101 was recognized as the risk factor for survival in several cancers including HCC (Fig. 1A). Then, integrating WGCNA, and the LASSO-Cox algorithm, candidates were identified and a robust ATG101-related gene signature (ATS) was established (Fig. 1B). Patients were classified into high and low ATS groups based on the median ATS score. Following that, the ATS predictive value was assessed in validation cohorts (Fig. 1C). Additionally, clinical and molecular characteristics, genomic changes, and therapeutic response were analyzed and compared between the ATS-high and ATS-low patients (Fig. 1D).

### Characteristics of ATG101 in HCC

Pan-cancer analysis showed that 24 out of 31 cancers had considerably greater expression of ATG101 in tumor tissues (Supplementary Fig. S1A,B). Moreover, ATG101 expression is associated with immune infiltration in several cancers (Supplementary Fig. S1C,D).

Association between ATG101 expression with clinical parameters suggested the potential tumor marker of ATG101 in HCC (Supplementary Fig. S2A–I). For instance, ATG101 expression rises in tandem with the advancement of stage or grade in LIHC (Supplementary Fig. S2E,F). Higher expression of well-known unfavourable prognostic indicators in HCC, such as AFP, DCP1A, GPC3, MDK, and SPP1, was more likely to be associated with ATG101-high (Supplementary Fig. S2I).

Autophagy is one of the major cellular responses under stress^34^. As a component of autophagome, ATG101 expression is tightly correlated with autophagy activity in HCC (Supplementary Fig. S2J,K).

**Figure 2:**
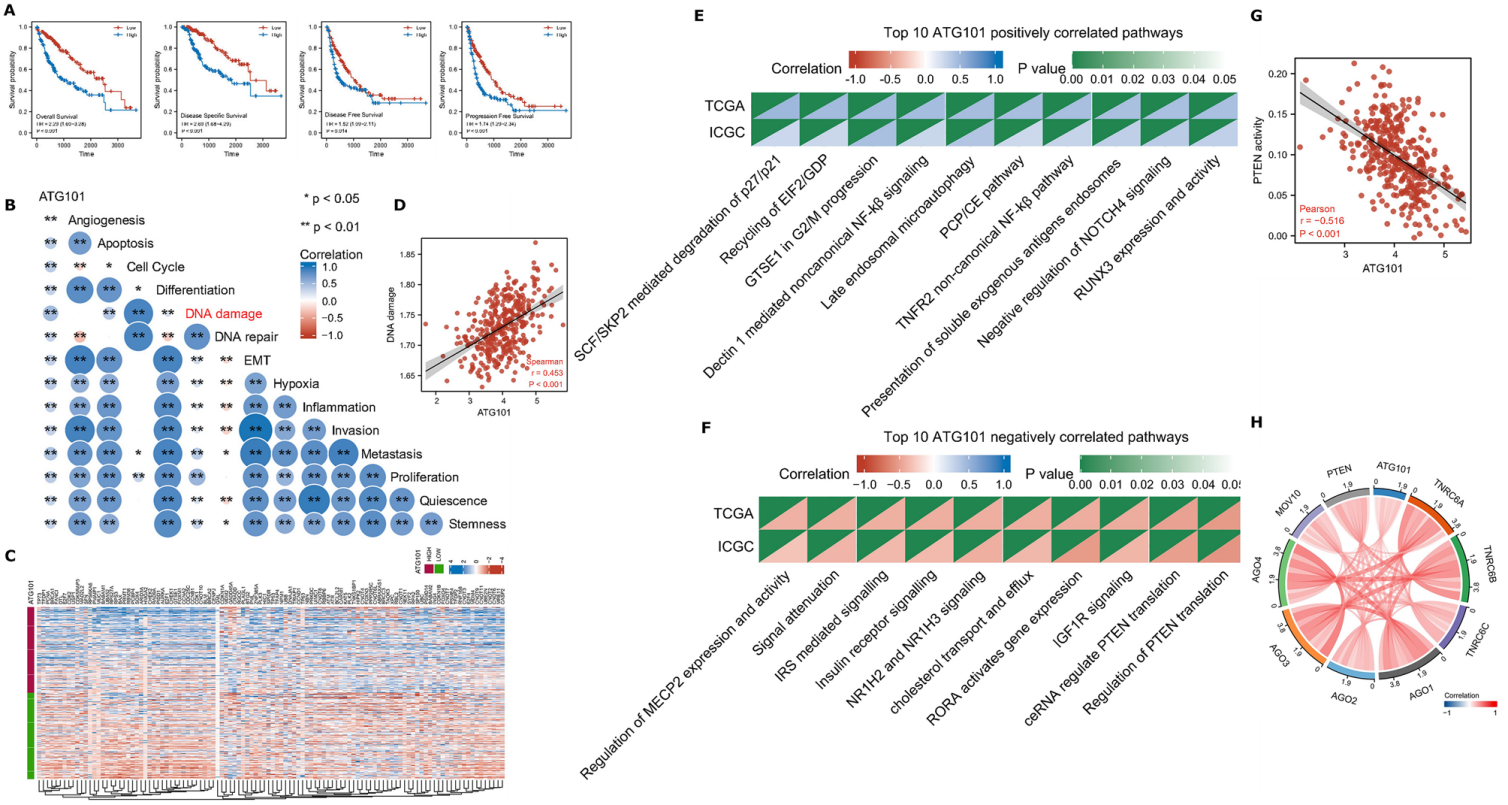
Cancer-related characteristics of ATG101 expression in HCC. (**A**) Survival analysis revealed that patients with increased ATG101 expression had worse OS, DSS, DFS, and PFI in LIHC. (**B**) Correlations between ATG101 expression and cancer hallmarks. (**C**) Heatmap of mark genes of DNA damage. (**D**) ATG101 expression is tightly linked to DNA damage. (**E**) Top 10 pathways positively associated with ATG101 expression. (**F**) Top 10 pathways negatively associated with ATG101 expression. (**G**) The regulation of PTEN translation pathway was shown to be strongly anti-correlated with ATG101. (**H**) Marker genes of the PTEN translation pathway.

Survival analysis revealed that high expression of ATG101 had worse OS, DSS, DFS, and PFI in the LIHC cohort (Fig. 2A). Next, ATG101 showed a significant association with all the cancer hallmarks (Fig. 2B). Particularly, ATG101 exhibits the strongest correlation to DNA damage (Fig. 2C,D).

To investigate the role of ATG101, we performed the correlation analysis between ATG101 expression and genome-wide pathway activities using Reactome genesets (Supplementary Table S1). ATG101 expression was significantly positively associated with cancer-associated biological processes such as the cell cycle, recycling of EIF2_GDP, NF-kB signaling, and autophagy, etc. (Fig. 2E). The top ATG101 negatively related pathways, on the other hand, were focused on metabolic activities such as NR1H2 and NR1H3 mediated signaling, insulin receptor signaling, and regulation of PTEN mRNA translation (Fig. 2F). Phosphatase and tensin homolog (PTEN) is a well-established tumor suppressor gene that inhibits cell proliferation and triggers apoptosis in a variety of human cancers. PTEN is also important in DNA damage response and repair, according to recent research^35^. Indeed, regulation of PTEN translation was shown to be most adversely linked with ATG101 expression (Fig. 2G,H). These findings point to ATG101 having an oncogenic potential in HCC.

### Establishment of an ATG101-related gene signature for prognosis

**Figure 3:**
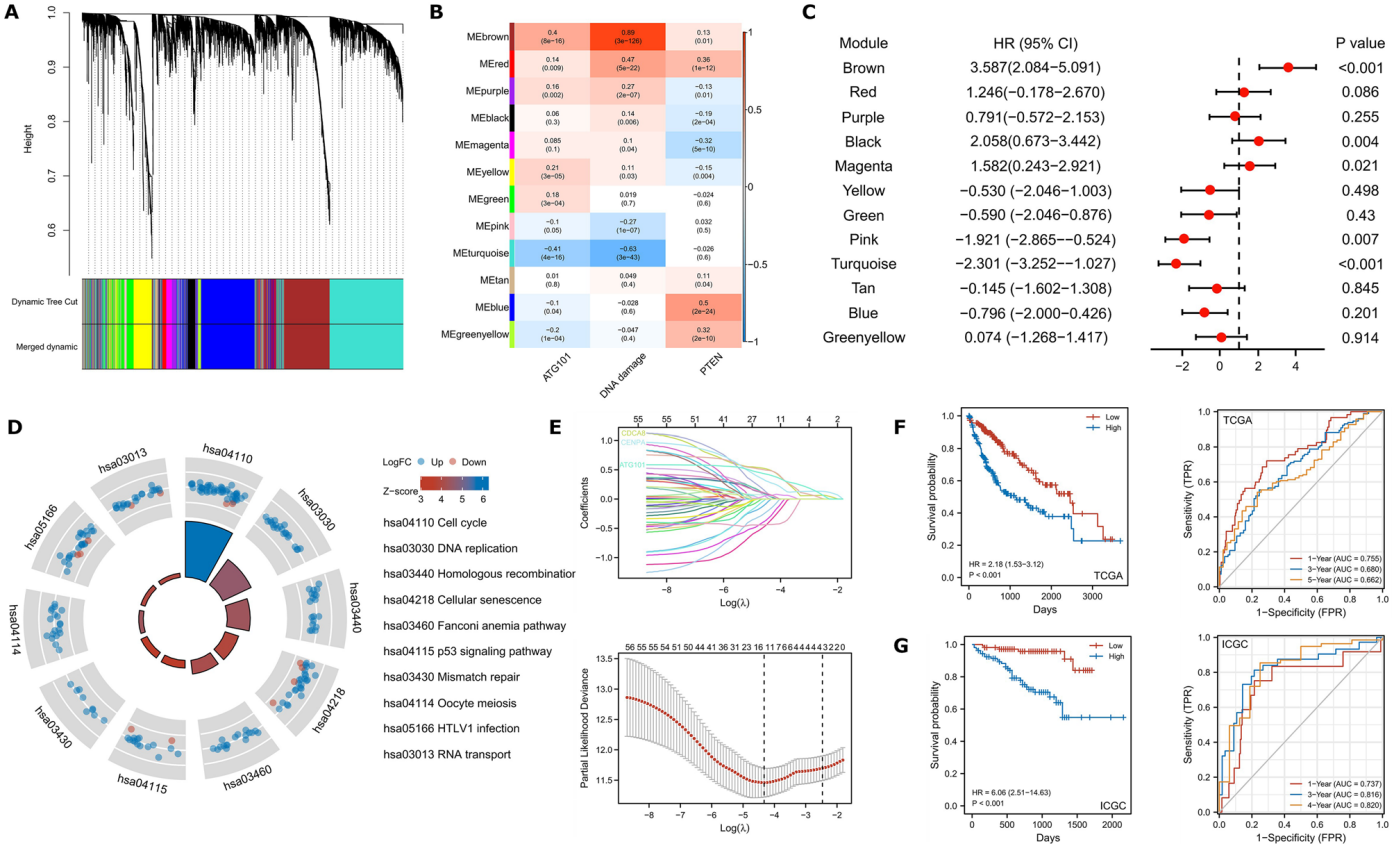
Establishment of an ATG101-related gene signature. (**A**) Modules were determined using WGCNA network. (**B**) Module-trait heatmap. (**C**) A forest plot depicting the prognosis of ME from each module. (**D**) KEGG enrichment of the brown module. (**E**) LASSO coefficient profiles of the expression of 51 candidate genes (upper panel), and the selection of the penalty parameter (λ) in the LASSO model (bottom panel). (**F**,**G**) Kaplan–Meier curves and time-dependent ROC plot of ATS in TCGA and ICGC cohorts, respectively.

WGCNA was performed to probe the ATG101-related molecular organization. To establish a scale-free co-expression network, a total of 12 non-grey modules were constructed with a power of = 5 as the best soft threshold (Fig. 3A). The brown module with the strongest correlation with ATG101 (r = 0.89, p = 3e−126) was considerably enriched in the cell cycle and DNA replication processes, as well as being linked to worse survival (Fig. 3B–D). Hub genes identified from the brown module were subjected to the LASSO-Cox regression analysis to find the most reliable prognostic signals. To avoid over-fitting, the optimal λ value of 0.0848 chosen (Fig. 3E). Finally, the following formula was devised: ATS = (0.06425)*CDCA8 + (0.1295)*CENPA + (0.0502)*ATG101. Between the high- and low-ATS groups, the Kaplan–Meier curves revealed a substantial difference in OS. Those who scored lower had a better chance of survival (Fig. 3F,G).

To enhance the prognostic potential of the model, risk score and AJCC stage were included into the nomogram (Supplementary Fig. S3A). The calibration plot revealed that the observed result and expected probability were quite similar (Supplementary Fig. S3B). The nomogram's AUCs for 1-, 3-, and 5-year OS predictions were 0.78, 0.75, and 0.73, respectively (Supplementary Fig. S3C). The prognostic nomogram had a better net benefit than the staging technique, according to the DCA analysis. These findings were confirmed using the ICGC cohort (Supplementary Fig. S3D).

### Genomic and molecular implications of ATS

**Figure 4:**
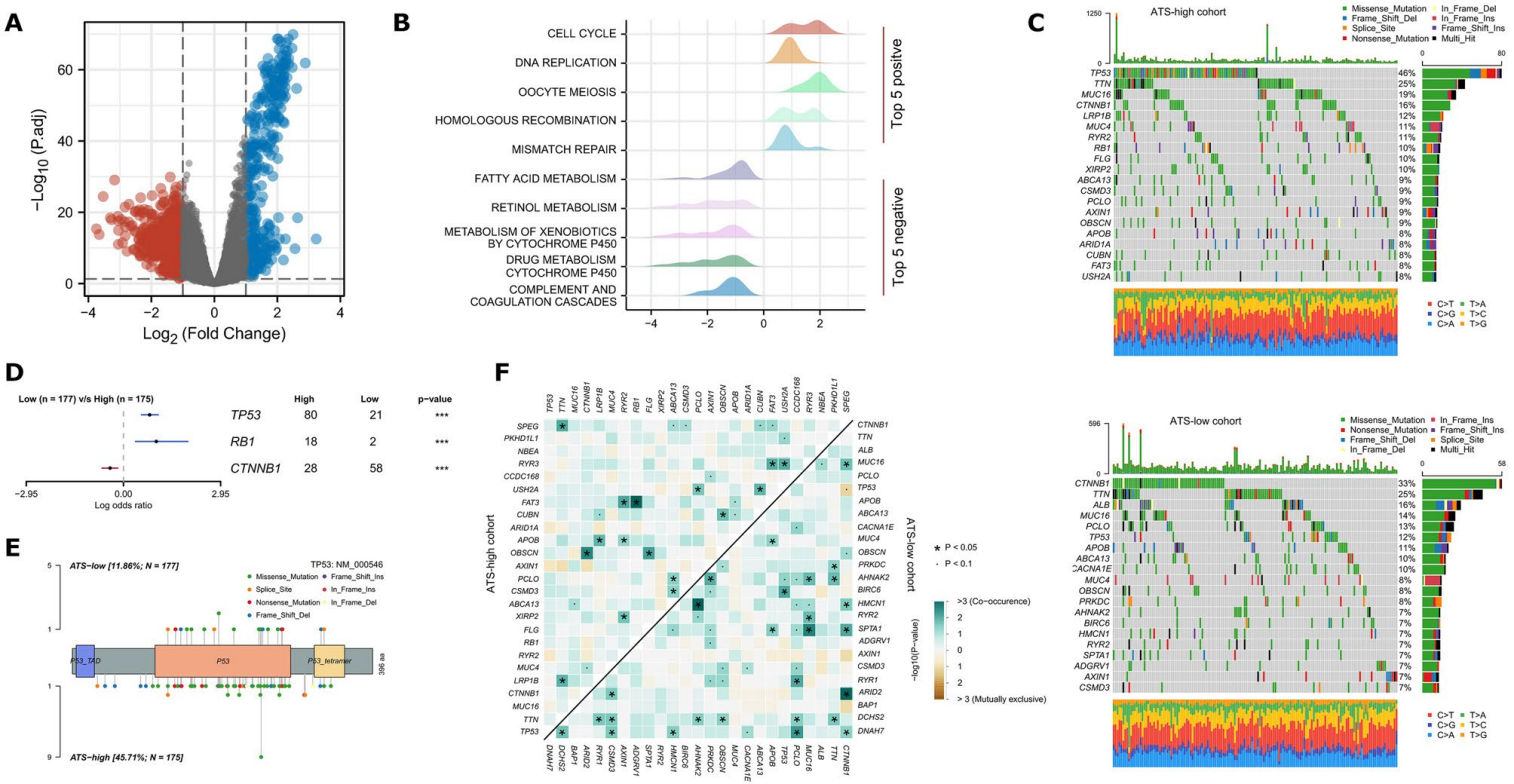
Comprehensive analyses of genomic alterations and biological process of ATS. (**A**) DEGs between ATS-low and ATS-high groups. (**B**) GSEA plot showing the enriched gene sets in ATS subgroups. (**C**) Mutation profiles of the top 20 most commonly mutated genes in ATS subgroups. (**D**) Prognosis effect of the differently mutated genes. (**E**) A lollipop plot demonstrating the differences in TP53 mutation locations between two cohorts. (**F**) The heatmap illustrating the co-occurrence and mutually exclusive mutations of the top frequently mutated genes.

Between ATS-high and ATS-low group, 331 substantially upregulated genes and 500 significantly downregulated genes were identified using a |logFC| ≥ 1 and FDR < 0.05 threshold (Fig. 4A). According to GSEA enrichment analysis, ATS-high HCCs were mostly enriched in cell cycle, DNA replication, and DNA repair pathways, while ATS-low HCCs were primarily enriched in different metabolic activities (Fig. 4B).

When applying the signaling network open resource to find the binary causal relationships among ATS-related DEGs^36^, multiple key nodes were disclosed (Supplementary Table S2). For instance, CDK1^37^ acts as the significant protein regulator that is up-regulated by genes such as RB1, NCOA3, PTTG1, etc., and up-regulates the activity of CSNK2A1, CSNK2B, ECT2, EZH2, PRC1, NPM1, thus resulting in processes such as the cell cycle (Supplementary Fig. 4).

Figure 4C showed the top 20 frequently mutated genes in ATS-high or low group respectively. Prognosis effect of the differently mutated genes, such as TP53, was also demonstrated (Fig. 4D). Figure 4E showed the distinct TP53 mutation locations between the two group using a lollipop plot. In addition, co-occurrence and mutually exclusive mutations were investigated, and in the ATS-low cohort, a unique case of TP53-CTNNB1 mutually exclusive mutation was discovered (Fig. 4F), indicating a shared effect induced by their respective mutations and the selective advantage of maintaining multiple copies of the mutations. These results further support the oncogene meaning of ATS.

### The ATS signature and the inflamed tumor microenvironment

**Figure 5:**
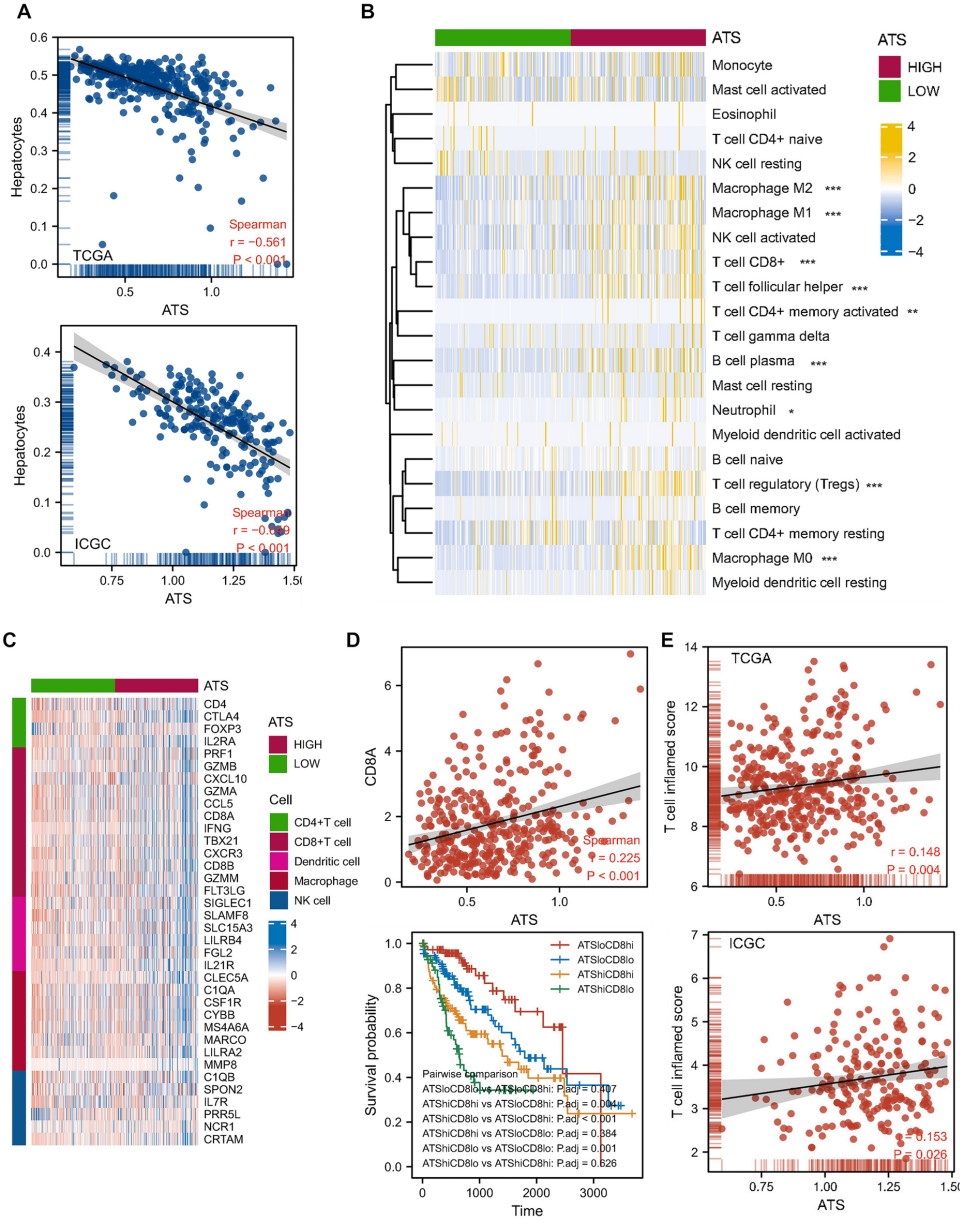
The ATS signature is associated with an inflamed TME. (**A**) Correlations between ATS and the hepatocyte enrichment. (**B**) TME cell proportions in ATS subgroups. (**C**) Positive correlation between the ATS signature and marker genes of immune cells. (**D**) Correlation between the ATS signature and CD8A expression, and the ATS-associated CD8+ T cell survival plot. (**E**) Correlations between ATS and TIS score in TCGA and ICGC cohorts, respectively.

Hepatocyte enrichment score was computed by xCell algorithm^25^. A negative correlation between ATS and hepatocyte enrichment was observed (Fig. 5A), indicating the loss of normal liver function. We also employed CIBERSORT to identify the immune cell types that infiltrate tumors. The percentage of CD4+ T cells, CD8+ T cells, myeloid dendritic cells, and macrophages differed between the ATS-high and ATS-low groups (Fig. 5B). Similarly, the effector genes of these tumor infiltrating immune cells (TIICs) were favorably linked with ATS (Fig. 5C).

The inflamed tumor microenvironment phenotype had a high CD8 positive rate^38^. Indeed, the ATS signature was strongly linked with CD8A expression (Fig. 5D). ATS-high/CD8+ T-low subpopulations were related with poor prognosis in HCC patients by K-M analysis and log-rank test (Fig. 5D). A TIS score quantifies an adaptive immune response that is activated but suppressed within the TME. We discovered a significant correlation between the TIS and the ATS signature (Fig. 5E), implying that ATS is linked with an inflamed phenotype.

### The ATS signature and the ICB-related genes

**Figure 6:**
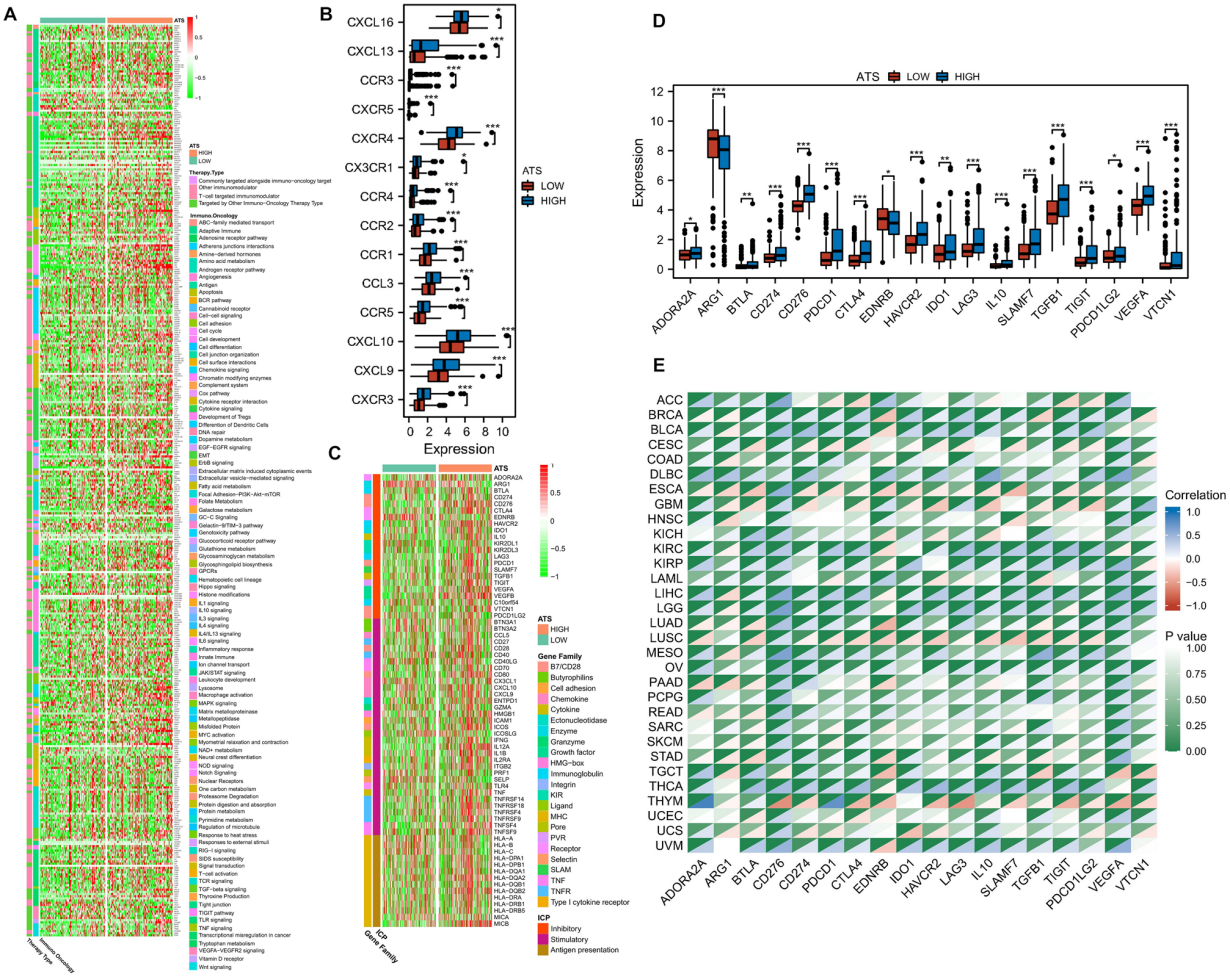
The ATS signature is associated with a diverse array of immunomodulators. (**A**) Positive correlation between the ATS signature and the immunomodulators. (**B**) Differences in the expression of chemokines between ATS-high and ATS-low groups. (**C**) Positive correlation between the ATS signature and ICB genes. (**D**) Differences in the expression of ICB genes between ATS-high and ATS-low states. (**E**) Correlation between the ATS signature and ICB-related genes in the TCGA pan cancer cohorts.

A broad spectrum of immunomodulators have been shown to be positively associated with ATS (Fig. 6A, Supplementary Fig. S5). For example, the recruitment of CD8+ T cells into the TME in HCC is induced by the key chemokines such as CXCL9, CXCL10, and CCR3, which were elevated in the high-ATS group (Fig. 6E). Furthermore, the ATS signature was favorably linked with the expression of MHC class I/II and antigen binding molecules (Supplementary Fig. S5), indicating an increase in antigen presentation.

Further, the ATS was positively correlated with the expression of multiple ICB-related genes (Fig. 6B). Expression of ICB genes such as PDCD1 (PD1), CD274 (PD-L1), CTLA4, HAVCR2, and TIGIT were elevated in the ATS-high group (Fig. 6C). In general, the critical regulatory variables involved in immunity work similarly in diverse tissues. We discovered that the positive correlation between ATS and ICB-related genes was evident not only in liver cancer, but also in other cancer types (Fig. 6D). Given PD1 as an example, ATS is positively correlated with PD1 expression in BRCA, BLCA, COAD, KIRC, KIRP, LIHC, LGG, LUAD, OV, TGCA, THYM, and UVM, while only negatively correlated with PD1 expression in CESC, ESCA and LUSC.

### The ATS signature is associated with exhausted T phenotype

**Figure 7:**
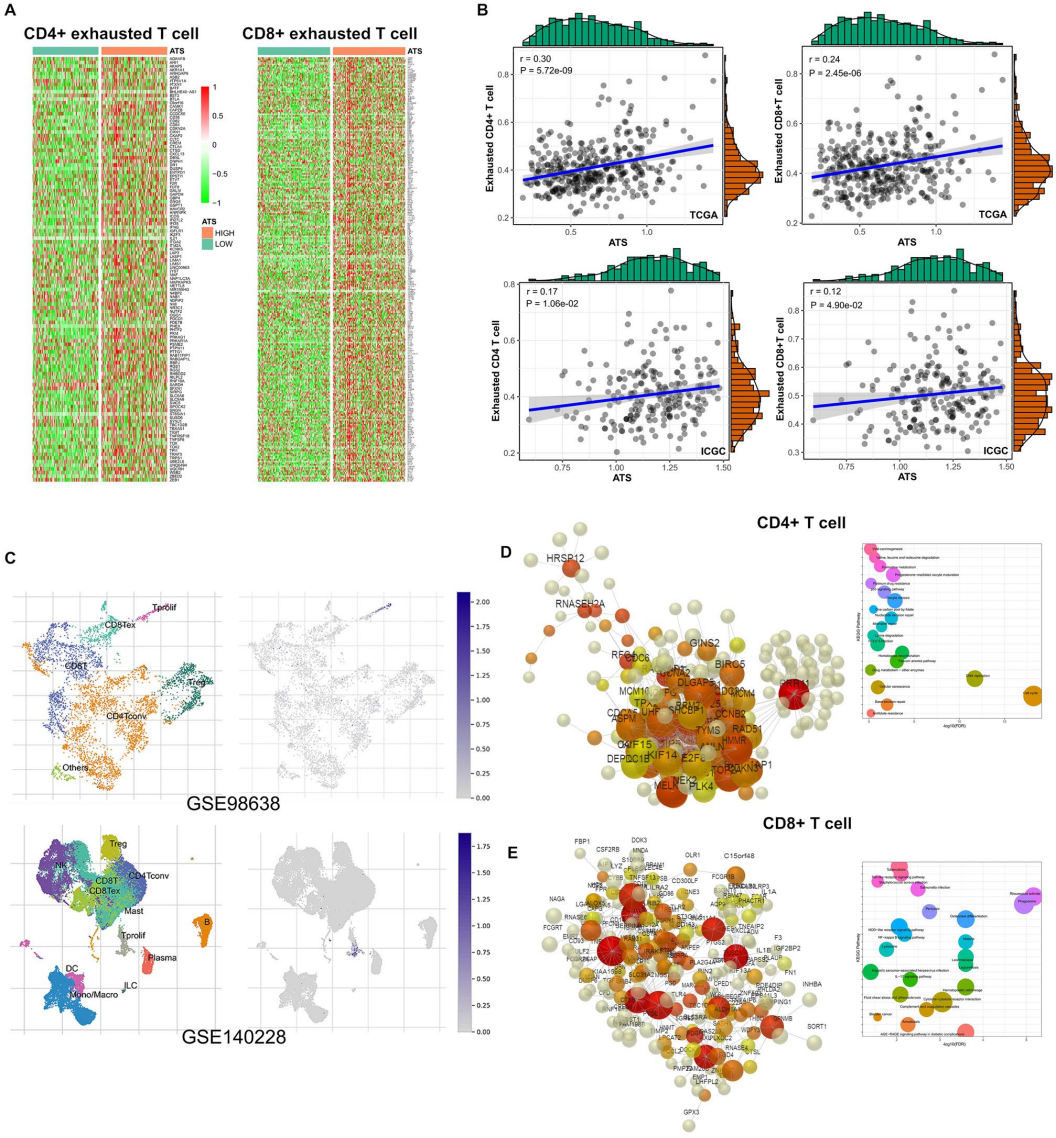
Impact of the ATS signature on T cells. (**A**) Marker gene distribution in exhausted CD4+ T cells and CD8+ T cells, respectively. (**B**) Correlation analysis of the ATS and the activity of exhausted CD4+ T cells or CD8+ T cells. (**C**) The distribution of ATS in different cell types using single-cell resolution. (**D**,**E**) ATS-related DEGs co-expression network and enrichment analysis in CD4+ and CD8+ T cells, respectively.

Deciphering phenotypic exhaustion in the immune subsets will be crucial to understanding collective immune dysfunction. As expected, the ATS signature was positively correlated with the activity of exhausted CD8+ and CD4+ T cells, respectively (Fig. 7A,B).

We next employed HCC single-cell sequencing data sets (i.e., GSE98638, and GSE140228) to locate the ATS signature within the cells, and discovered that proliferative T cells clearly expressed the ATS signature (Fig. 7C).

To explore the significance of this phenomenen, the ATS-related DEGs were then used in an enrichment analysis with Immuno-Navigator, a database for gene co-expression in specific immune cells^39^. Within CD4+ T cells, DEGs were significantly enriched in pathways such as cell cycle, DNA replication, homologous recombination, Fanconi anemia pathway, meiosis, nucleotide excision repair, base excision repair, and p53 signaling pathway (Fig. 7D), implying that the ATS signature has proliferative implications in CD4+ T cells. Within CD8+ T cells, the ATS signature was linked to the terms Rheumatoid arthritis, Phagosome, Malaria, Hematopoietic cell lineage, Leishmaniasis, AGE-RAGE signaling pathway in diabetic complications, osteoclast differentiation, Legionellosis, and Cytokine-cytokine receptor interaction(Fig. 7E), implying that the ATS signature has a chronic immune-inflammation implication in CD8+ T cells.

### Therapy responsiveness of the ATS signature

**Figure 8:**
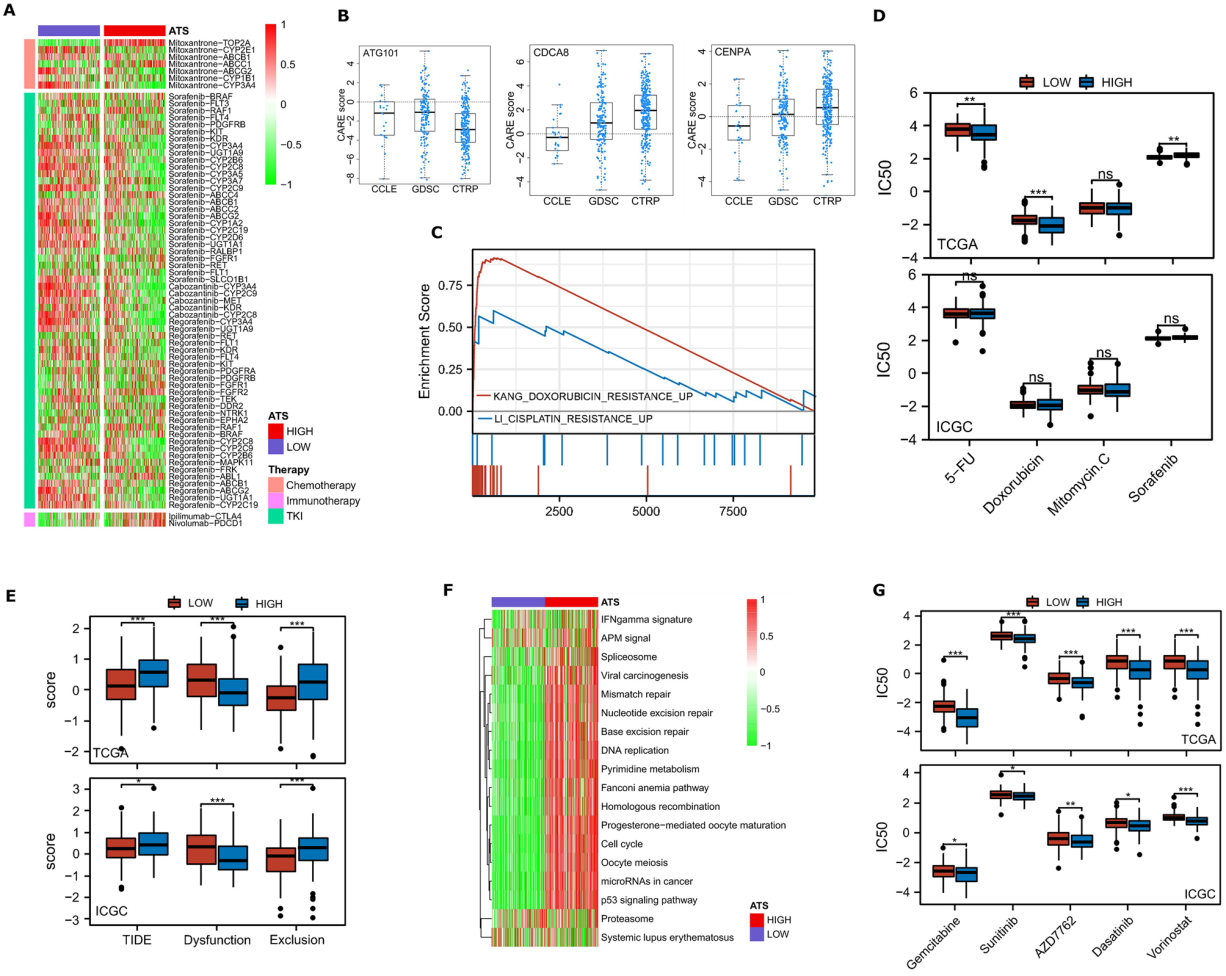
The ATS signature acts as a promising marker for different therapies. (**A**) Correlation between ATS and HCC-related pharmacological targets identified in the Drugbank database. (**B**) ATS-related durg resistance as assessed by CARE. (**C**) GSEA predicted an association between the gene signature and treatment resistance. (**D**) The connections between IC50 values of commonly used anti-HCC compounds and ATS state. (**E**) The ICB response calculated using TIDE algorithm. (**F**) Differences in immunotherapy-predicted pathway enrichment scores between high- and low-ATS groups. (**G**) Chemotherapy sensitivity differences between high-risk and low-risk groups.

After screening the Drugbank database, the results revealed that the low-ATS group had a better response to chemotherapy and targeted treatment (Fig. 8A). Considering tumors always promote resistance to therapy, we used the CARE algorithm to analyze the ATS signature. Our results suggested that ATS gene expression, particularly ATG101, is linked to drug resistance (Fig. 8B). Furthermore, GSEA indicated that greater ATS expression is strongly linked with resistance to drugs like doxorubicin and cisplatin based on the changed gene sets of various pharmacological treatments retrieved from MSigDB (Fig. 8C). The chemotherapeutic sensitivity of widely used drugs like sorafenib was not consistent between ATS subgroups (Fig. 8D).

Despite the fact that increased expression of PD1 and CTLA4 in the ATS-high group supporting immunotherapy effectiveness, ATS-low patients had lower TIDE scores, indicating that they were more receptive to immunotherapy than ATS-high patients (Fig. 8E). Furthermore, the ATS-high group had higher enrichment scores for immunotherapy-predicted pathways (Fig. 8F). This re-validated the finding that ATS was negatively correlated with the response to ICB.

Thus, the chemotherapeutic sensitivity was calculated using agents frequently utilized in the cancer field. In comparison to the ATS-low group, patients in the ATS-high group had significantly lower IC50 values for the drugs such as Gemcitabine (DNA synthesis inhibitor), Sunitinib (RTK inhibitor), AZD7762 (CHEK inhibitor), Dasatinib (Src inhibitor), and Vorinostat (HDAC inhibitor) (Fig. 8G), indicating a potential treatment sensitivity of these patients to the aforementioned drugs. We infer from these findings that the ATS-derived signature has the potential to predict immunotherapy response and targeted therapy sensitivity.

## Discussion

To date, autophagy signature for prognosis have been identified in several cancer types, including breast^40^, glioma^41^, and colorectal cancer^42^. This study, for the first time, established a prognostic model using autophagy-related molecules and disclose its implication in HCC.

First, we identified ATG101 as one of the key risk factors for prognosis in HCC. Among various hallmarks of cancer, ATG101 expression is mostly correlated with DNA damage. Indeed, understanding the mechanisms underlying DNA damage-induced cellular autophagy is critical for fully exploiting the anti-cancer potential of DNA-damaging agents^34^. ATG101 expression is largely anti-correlated with the PTEN pathway. These results give clues on the potential mechanism of ATG101 in HCC since DNA damage surveillance systems and their links to the PTEN/PI3K/Akt signaling pathway regulate DNA repair during cell growth activation^43^.

Different bioinformatic and statistical methods were combined to construct a robust ATS signature for prognosis, and confirmed in independent cohorts. Indeed, genes within ATS have been studied previously. For instance, CDCA8 knockdown inhibits HCC development and stemness by restoring the ATF3 tumor suppressor and inactivating AKT/β-catenin signaling^44^. As a mitotic gene, the prognostic implication of CENPA has been discribed in HCC recently^45^.

DNA damage is linked to a pro-inflammatory secretory phenotype that helps reshape the tumor-immune microenvironment. As a result, the levels of infiltration of many effector TIICs, including CD8+ T cells, CD4+ T cells, macrophages, and dendritic cells, rose significantly in the high-ATS group. Upregulation of immunological checkpoints such PD-L1/PD-1, which is triggered by pre-infiltrating TIICs, is another important feature of an inflamed TME. Immunological checkpoints decrease pre-existing cancer immunity to prevent an overreaction, but they also cause immune evasion. Furthermore, the enrichment of immunotherapy-predicted pathways and the TIS score were both positively associated to ATS. Thus, ATS status represents an inflamed yet exhausted TME.

Because of the increased T cell exclusion, TIDE evaluation indicated that HCC with high-ATS was not susceptible to ICB. However, we discovered that ATS may have an immunosuppressive effect by downregulating the expression of key chemokines such as CXCL9, CXCL10, and CXCR3, and therefore decreasing the cancer immunity. Notably, the enrichment scores of exhausted CD8+ or CD4+ cells were substantially correlated with ATS. Increased expression of inhibitory markers and a gradual and hierarchical loss of function define exhausted T cells. Despite the fact that cancer-induced exhaustion in CD8+ T cells has been well documented and recognized as a therapeutic target, new research reveals that CD4+ T-cell exhaustion is common in cancer^46^. Because ATS-related CD4+ T cells have been linked to a variety of cell cycle-related activities, proliferation-exhaustion is likely to be clinically relevant and warrants further investigation.

Furthermore, the CARE and TIDE algorithms indicate that ATS is linked to drug resistance to targeted therapy and immunotherapy, indicating that the gene signature might be a useful predictor of therapeutic resistance in HCC patients. Alternative therapy options for HCC patients, especially those with high-ATS, are critically required in this regard. Several drugs, including tyrosine kinase inhibitors, DNA repair inhibitors, and HDAC inhibitors, were shown to have potential applicability in the drug sensitivity assessments. A combination of targeted therapy and immunotherapy might be suitable for patients with higher ATS.

This study has several limitations. First, further experimental research is required to clarify the biological roles of ATG101 that underpin the gene signature. Second, since this is a retrospective research, the ATS predictive robustness and clinical utility will need to be confirmed in prospective trials.

To conclude, we developed an ATG101-related gene signature to identify high-risk HCC patients. ATS was found to be significantly associated with exhausted CD8+ or CD4+ cells. The ATS signature-based approach may be a valuable tool for identifying high-risk individuals who may benefit from anti-HCC combination therapy.

## Supplementary Information

Supplementary Figures.

Supplementary Table 1.

Supplementary Table 2.

## Data Availability

The datasets generated and analyzed during the current study are available in the TCGA data source (https://xena.ucsc.edu) and ICGC data portal (https://dcc.icgc.org/). All data and R script in this study are available from the corresponding author on reasonable request.

## Abbreviations

HCC: Hepatocellular carcinoma
ICB: Immune checkpoint blockade
TME: Tumor microenvironment
PD-1: Programmed cell death protein 1
PD-L1: PD-1 ligand 1
CTLA-4: Cytotoxic T-lymphocyte-associated protein 4
TIDE: Tumor immune dysfunction and exclusion
OS: Overall survival
PFI: Progression free survival
DSS: Disease specific survival
DFS: Disease free survival
TCGA: The cancer genome atlas
Cox-PH: Cox proportional-hazards
LASSO: Least absolute shrinkage and selection operator
ssGSEA: Single-sample gene set enrichment analysis
tROC: Time-dependent receiver operating characteristic
DCA: Decision curve analysis
TISCH: Tumor immune single-cell hub
TIS: T cell inflamed score
WGCNA: Weighted gene co-expression network analysis
DEG: Differential expressed genes
IC50: Half-maximal inhibitory concentration
CTRP: Cancer therapeutics response portal
CCLE: Cancer cell line encyclopedia
GDSC: Genomics of drug sensitivity in cancer databases
CARE: Computational analysis of resistance
LGG: Brain lower grade glioma
LIHC: Liver hepatocellular carcinoma
MESO: Mesothelioma
ACC: Adrenocortical carcinoma
BLCA: Bladder urothelial carcinoma
BRCA: Breast invasive carcinoma
CESC: Cervical squamous cell carcinoma and endocervical adenocarcinoma
CHOL: Cholangiocarcinoma
COAD: Colon adenocarcinoma
HNSC: Head and neck squamous cell carcinoma
KIRC: Kidney renal clear cell carcinoma
KICH: Kidney chromophobe
LAML: Acute myeloid leukemia
LUAD: Lung adenocarcinoma
LUSC: Lung squamous cell carcinoma
MESO: Mesothelioma
OV: Ovarian serous cystadenocarcinoma
PAAD: Pancreatic adenocarcinoma
PCPG: Pheochromocytoma and paraganglioma
PRAD: Prostate adenocarcinoma
READ: Rectum adenocarcinoma
SARC: Sarcoma
SKCM: Skin cutaneous melanoma
STAD: Stomach adenocarcinoma
TGCT: Testicular germ cell tumors
THCA: Thyroid carcinoma
THYM: Thymoma
UCEC: Uterine corpus endometrial carcinoma
UCS: Uterine carcinosarcoma
UVM: Uveal melanoma
