# Supplementary material for: ATG101-related signature predicts prognosis and therapeutic option in hepatocellular carcinoma
Source: Sci Rep. 2022 Oct 27;12:18066. doi: 10.1038/s41598-022-22505-5 (PMC9613769; doi:10.1038/s41598-022-22505-5)
Supplement: Supplementary file 1 — Supplementary Figures. [file 41598_2022_22505_MOESM1_ESM.docx]

**Supplementary figures**


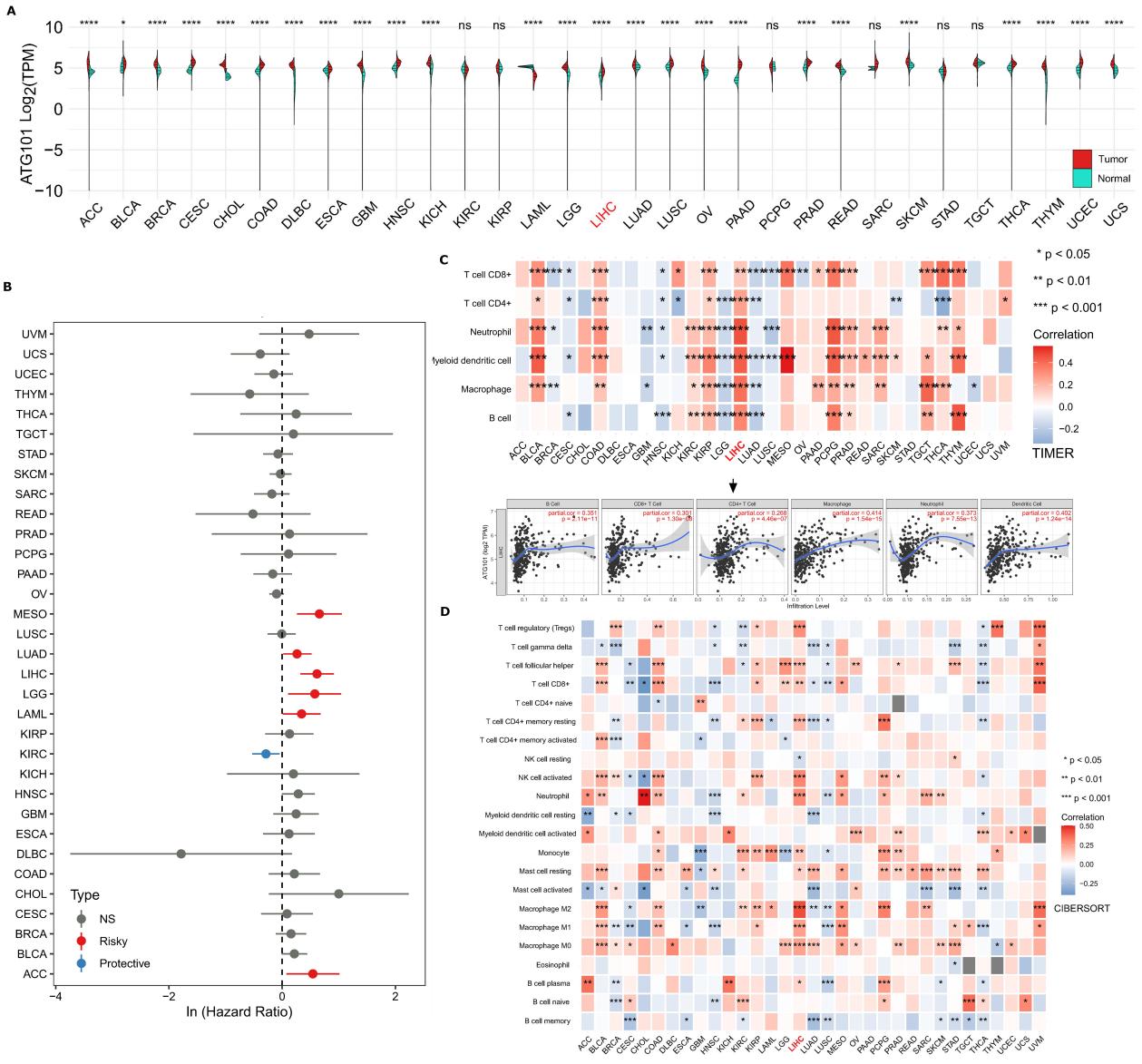


**Fig.S1** ATG101 expression in pan-cancer. (**A**) Differential expression of ATG101 between tumor and normal tissues in pan-cancer. (**B**) Analysis of ATG101 expression in pan-cancer using univariate Cox regression. (**C**) Correlation between ATG101 expression and immune cell infiltration using TIMER method. (**D**) Estimating immune cell infiltration Using the Cibersort algorithm.


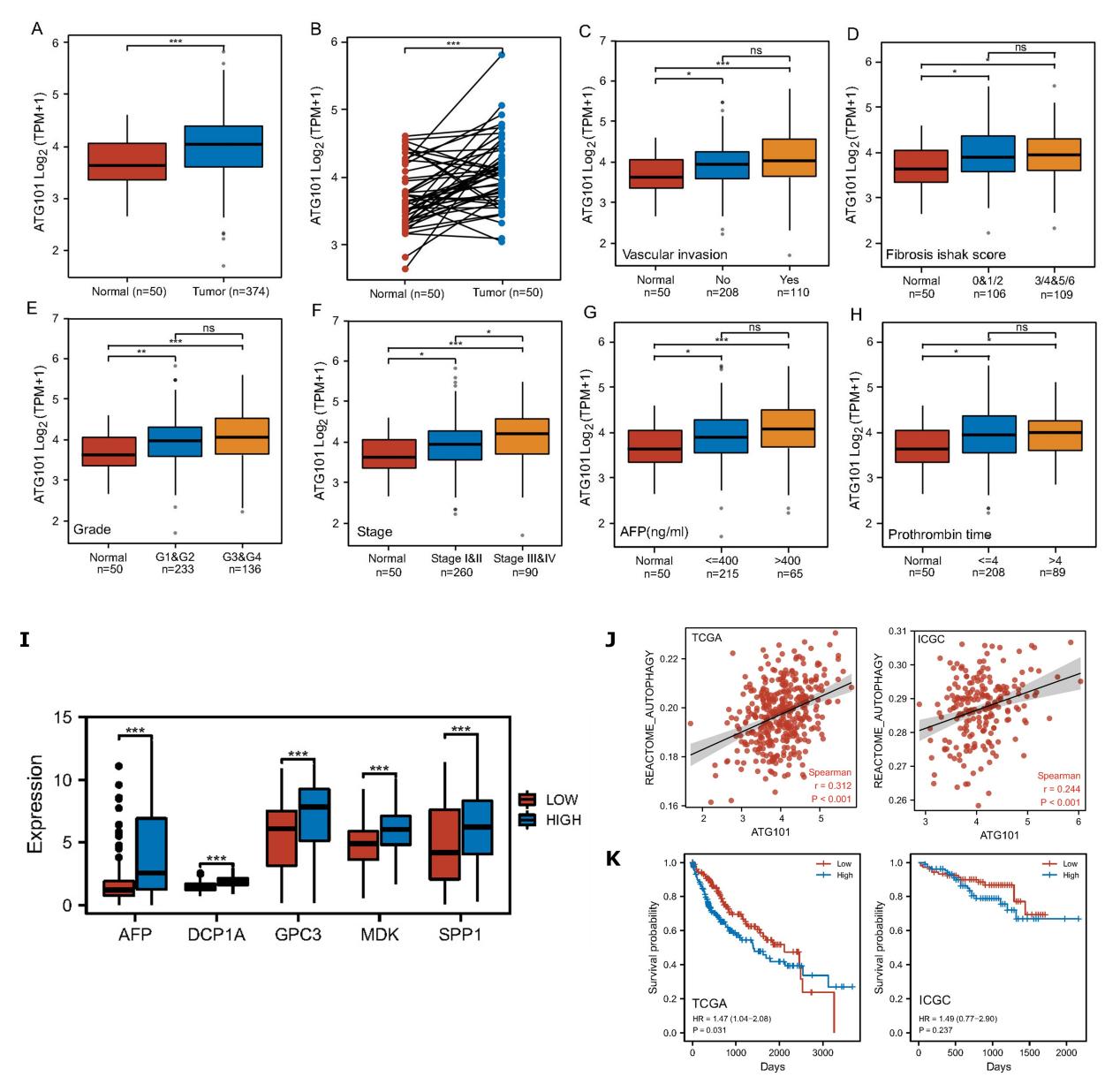


**Fig.S2** ATG101 expression in LIHC. (**A**) Expression of ATG101 between tumor and normal tissues. (**B**) Paired expression of ATG101 between tumor and normal tissues. (**C-H**) ATG101 expression in subgroups of clinical characteristics including vascular invasion (C), fibrosis (D), grade (E), stage (F), AFP concentration (G), and prothrombin time (H). (**I**) Differences in the expression of well-known HCC biomarkers between ATG101 subgroups. (**J**) ATG101 expression is tightly linked to autophagy activity in TCGA and ICGC cohort, respectively. (**K**) Prognosis effect of autophagy activity in TCGA and ICGC cohort, respectively.


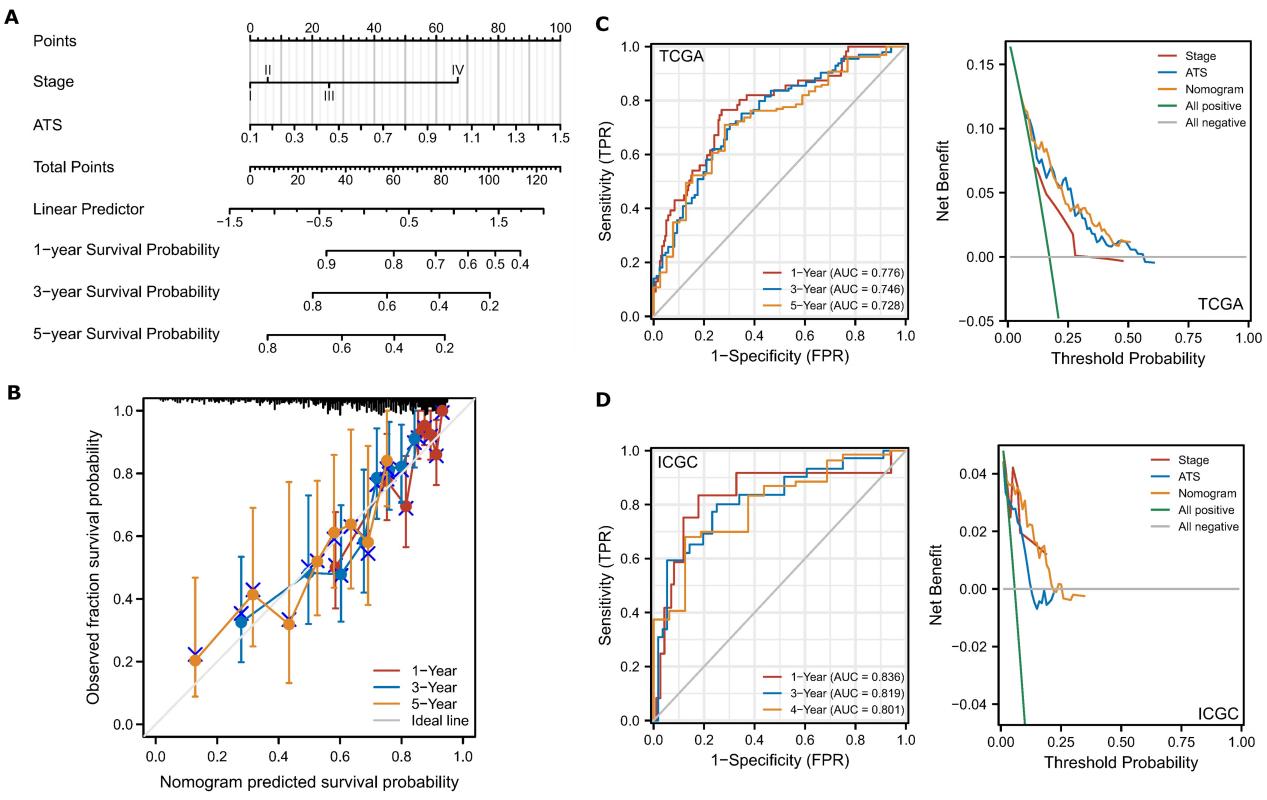


**Fig.S3** Construction of a clinical predictive nomogram. (**A**) The hybrid nomogram combining the ATS score with the AJCC stage. (**B**) Calibration curves show the consistency between the nomogram-predicted OS probability and the observed OS probability. (**C-D**) The AUCs of the time-dependent ROC curves (left) and DCA curves (right) illustrating the net benefit of the combined nomogram compared with other individual models in TCGA and ICGC cohort, respectively.


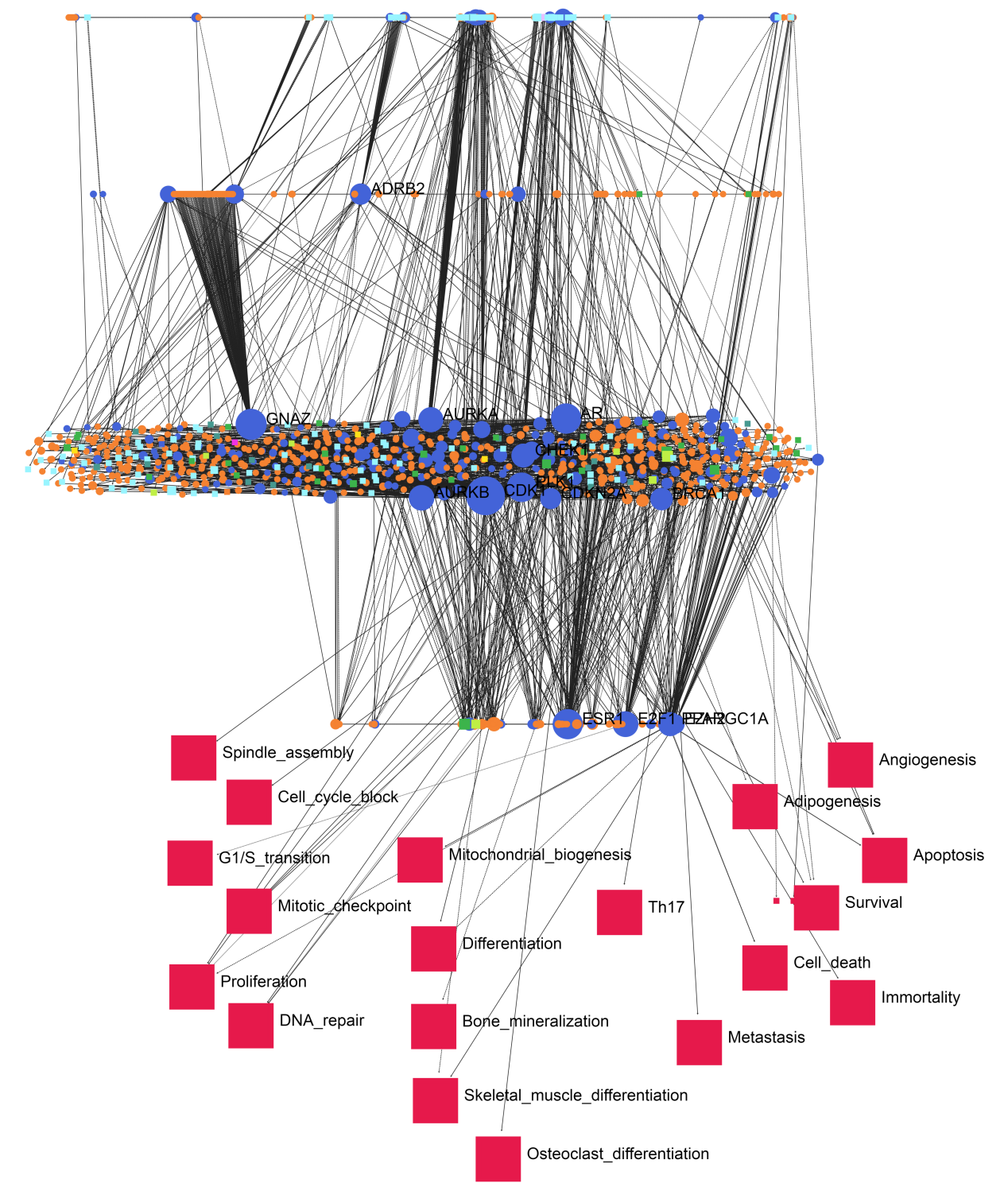


**Fig.S4** ATS-related signaling network. The signed directed network (1109 nodes and 1554 edges) determine the signaling cascade to the effect and mechanism output.


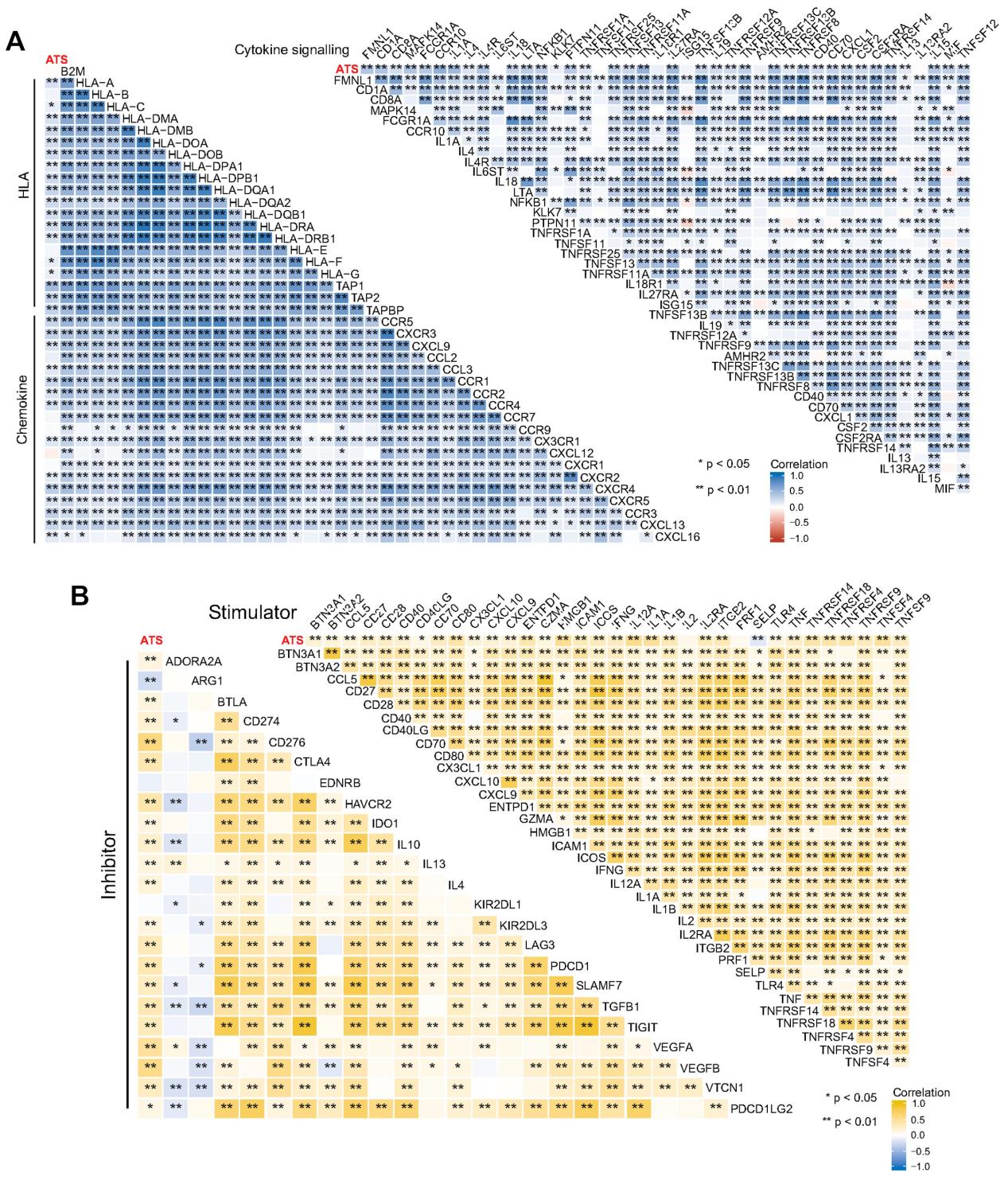


**Fig.S5** The ATS signature is associated with immune modulators. (**A**) Correlation between the ATS signature and cytokines or chemokines. (**B**) Correlation between the ATS signature and ICB-related genes.
